# Sodium-Glucose Co-Transporter 2 Inhibitors for Non-Alcoholic Fatty Liver Disease in Asian Patients With Type 2 Diabetes: A Meta-Analysis

**DOI:** 10.3389/fendo.2020.609135

**Published:** 2021-02-11

**Authors:** Chloe Wong, Clyve Yu Leon Yaow, Cheng Han Ng, Yip Han Chin, Yi Fen Low, Amanda Yuan Ling Lim, Mark Dhinesh Muthiah, Chin Meng Khoo

**Affiliations:** ^1^ Yong Loo Lin School of Medicine, National University of Singapore, Singapore, Singapore; ^2^ Department of Medicine, National University Hospital, Singapore, Singapore; ^3^ National University Centre for Organ Transplantation, National University Hospital, Singapore, Singapore

**Keywords:** hepatic fat, non-alcoholic fatty liver disease, sodium-glucose co-transporter-2 inhibitors, type 2 diabetes, meta-analysis

## Abstract

**Objective:**

Non-alcoholic fatty liver disease (NAFLD) is a very common disorder among patients with type 2 diabetes and may share causal relationship. Type 2 diabetes is a risk factor for progression and potential poor outcomes in NAFLD patients. This meta-analysis aimed to analyze the current evidence of sodium-glucose co-transporter-2 inhibitors (SGLT2i), a glucose-lowering drug to improve NAFLD in patients with Type 2 Diabetes.

**Methods:**

Medline, Embase and Cochrane Central Register of Controlled Trials were searched for articles examining efficacy of SGLT2i on treatments of NAFLD in type 2 diabetes in July 2020, and articles were sieved. Continuous data were extracted in the form of mean and standard deviation and were pooled with standardized mean difference (SMD).

**Results:**

10 articles involving 555 patients from seven randomized controlled trials (RCTs) and three cohort studies, were included in this meta-analysis. Our analysis revealed significant improvements in hepatic fat content (after treatment: -0.789 (-1.404 to -0.175), p = 0.012; compared with control: -0.923 (-1.562 to -0.285), p = 0.005), AST (After Treatment: -0.539 (-0.720 to -0.357), p < 0.001; compared with control: -0.421 (-0.680 to -0.161), p = 0.001), ALT (after treatment: -0.633 (-0.892 to -0.373), p < 0.001; compared with Control: -0.468 (-0.685 to -0.251), p < 0.001), body composition (BMI: after treatment: -0.225 (-0.456 to 0.005), p = 0.055; compared with Control: -1.092 (-2.032 to -0.153), p = 0.023), glycemic control (HbA1c: After Treatment: -0.701 (-1.098 to -0.303), p = 0.001; compared with control: -0.210 (-0.603 to 0.183), p = 0.295), lipid parameters (Triglycerides: after treatment: -0.230 (-0.409 to -0.052), p = 0.011; compared with control: -0.336 (-0.597 to -0.076), p = 0.011), inflammatory markers (serum ferritin: after treatment: -0.409 (-0.694 to -0.124), p = 0.005; compared with control: -0.814 (-1.688 to 0.059), p = 0.068) after SGLT2i treatment, and when compared against controls. There was a trend in the improvement in fibrosis markers after SGLT2i treatment.

**Conclusions:**

SGLT2i is an effective treatment to improve NAFLD among patients with type 2 diabetes. Further studies are needed to understand the direct and indirect effects of SGLT2i on NAFLD and if SGLT2i could prevent the progression of NAFLD or NASH. SGLT2i could potentially be considered for patients with type 2 diabetes and NAFLD, if there are no contraindications.

## Introduction

Non-alcoholic fatty liver disease (NAFLD) is the most common, but unappreciated liver disease in developed countries (1). It occurs when there is excessive fat accumulation in the liver without a history of significant alcohol consumption or other underlying causes which result in fat accumulation. NAFLD has two principle phenotypes, non-alcoholic fatty liver (NAFL), and non-alcoholic steatohepatitis (NASH), which are differentiated by the presence of inflammation and hepatocyte injury (2). NAFLD can subsequently progress to fibrosis and liver cirrhosis, and might lead to the development of liver cancer (3).

The rising prevalence of NAFLD mirrors the rising rates of obesity, and is closely associated with its complications of metabolic syndrome and type 2 diabetes (4–7). The current global prevalence of NAFLD is estimated about 25.24% (8). Among patients with type 2 diabetes, the prevalence of NAFL and NASH is as high as 55.5 and 37.3%, respectively (9). It has been shown that type 2 diabetes is an independent predictor for the progression of NAFL to NASH and liver fibrosis (10, 11). On the other hand, NAFLD is also associated with a higher risk of incident type 2 diabetes and diabetes-related complications, such as chronic kidney disease and retinopathy (12–14). With the global diabesity epidemic, it is projected that the prevalence of NAFLD and the cost of treating NAFLD will rise exponentially, with a study projecting the healthcare costs of NAFLD in United States to be $103 billion (15).

Sodium-glucose co-transporter 2 inhibitors (SGLT2i) are a relatively new class of glucose-lowering drugs, and has been shown to reduce mortality from cardiovascular disease, prevent hospitalization from heart failure and reduce progression of diabetic kidney disease (16). Several studies and meta-analyses have also shown that SGLT2i could improve liver steatosis (17, 18), but the focus is largely on canagliflozin. As such, the effects of the other SGLT2i treatments, such as dapagliflozin, empagliflozin, ipragliflozin and luseogliflozin remain largely unknown. While we believe that the effect of SGLT2i on NAFLD is of class effect, this remains speculative as there is no head-to-head comparison. This meta-analysis aimed to consolidate the current evidence of the effects of SGLT2i drugs on NAFLD among patients with type 2 diabetes.

## Material and Methods

### Search Strategy

This meta-analysis was conducted in accordance to the Preferred Reporting Items for Systematic Reviews and Meta-analyses (PRISMA) guidelines (19). Searches on electronic databases Medline, Embase and Cochrane Central Register of Controlled Trials were conducted on 21^st^ June 2020. Keywords and thesaurus terms pertaining to “NAFLD” and “SGLT2 inhibitors” were used in the search. References of relevant articles were also searched manually for additional studies. The search strategy is attached in the **Supplementary Material 1**.

### Selection Criteria and Eligibility Criteria

Articles identified from the search underwent a title and abstract sieve. A full-text review was then conducted independently by two authors and any discrepancies were discussed and addressed, until a full consensus was reached. The inclusion criteria for this meta-analysis is as follows: 1) a definite diagnosis of both NAFLD and Type 2 Diabetes in patients and 2) comparative studies comparing SGLT2i against anti-diabetic treatments. In addition, only original articles were included, excluding commentaries, conference abstracts, editorials and non-English studies.

### Data Extraction and Outcomes

Data extraction was conducted independently by two authors. The data extracted from the studies include the author, year of publication, country of study, study design, dosage of SGLT2i and control treatments, sample size, demographics (gender composition, age, duration of diabetes, concomitant medication usage, comorbidities) and relevant primary data. For continuous variables, the mean and standard deviation (SD) were extracted. In the case where mean and SD were not available, the data was transformed according to existing formulae; using calculations from Hozo et al. for conversion from median and range (20), and calculations from Wan et al. for conversion from median and interquartile range (21). The outcomes of this meta-analysis include changes in body composition, metabolic parameters, adipokines and inflammatory markers, steatosis markers, fibrosis markers, as well as liver and renal biomarkers.

### Statistical Analysis and Quality Assessment

To account for the different units of analysis, the standard mean differences was preferred in the analysis (22). Continuous data was pooled with standardized mean difference (SMD). All analysis was conducted in STATA and p < 0.05 was considered statistically significant. Quality assessment of cohort studies was conducted *via* the Newcastle Ottawa Scale (NOS) while risk of bias assessment of randomized controlled trials (RCT) was carried out using Cochrane’s Risk of Bias 2 (RoB2) tool. The RoB2 tool assesses quality on 5 domains primarily the randomization process, deviations from intended interventions, missing outcome data, outcome measurements and reporting (23). The NOS assesses quality based on three main domains including selection, comparability and outcome (24).

## Results

### Baseline Characteristics

**Figure 1:**
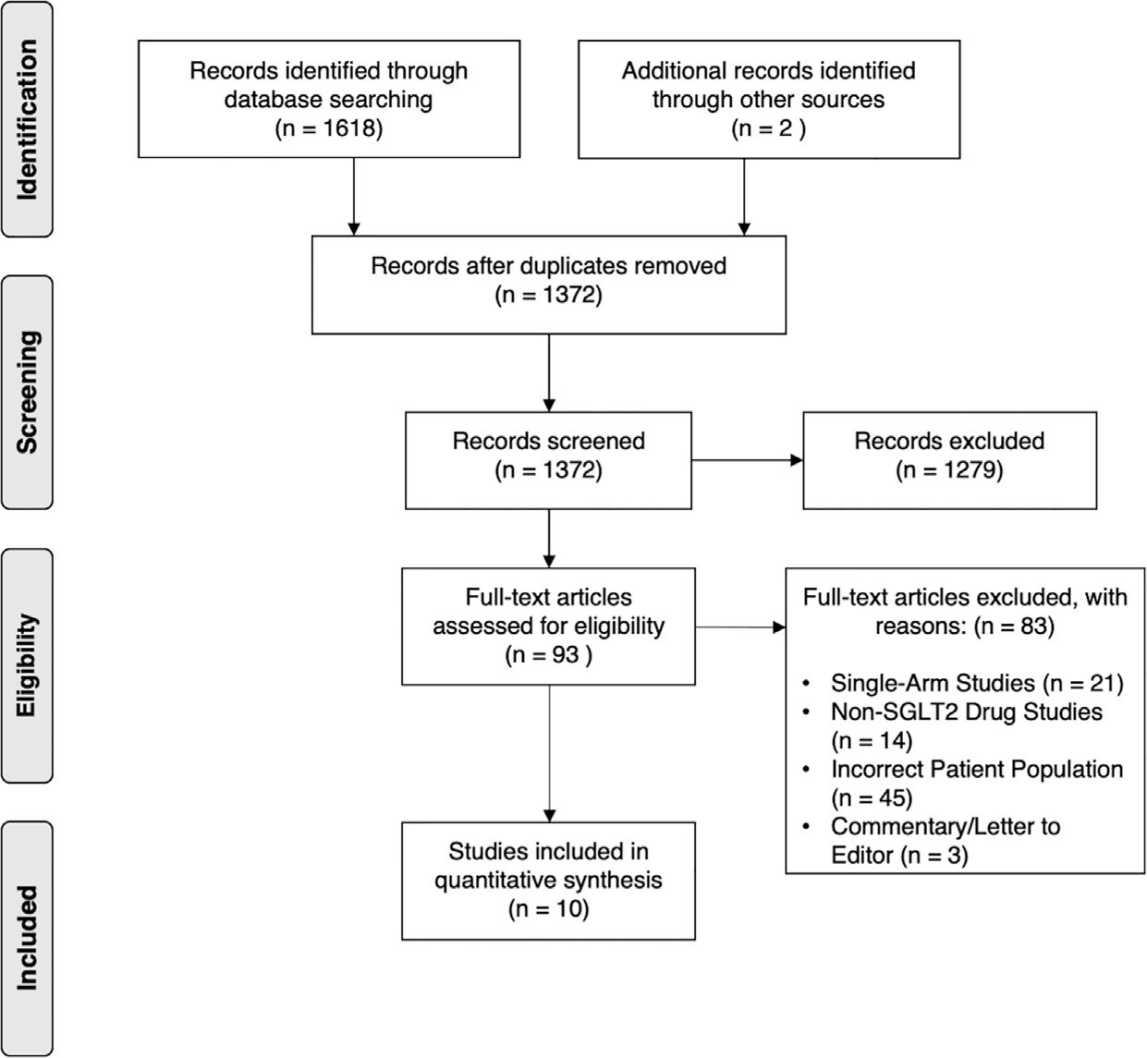
PRISMA Flow Diagram.

**Figure 2:**
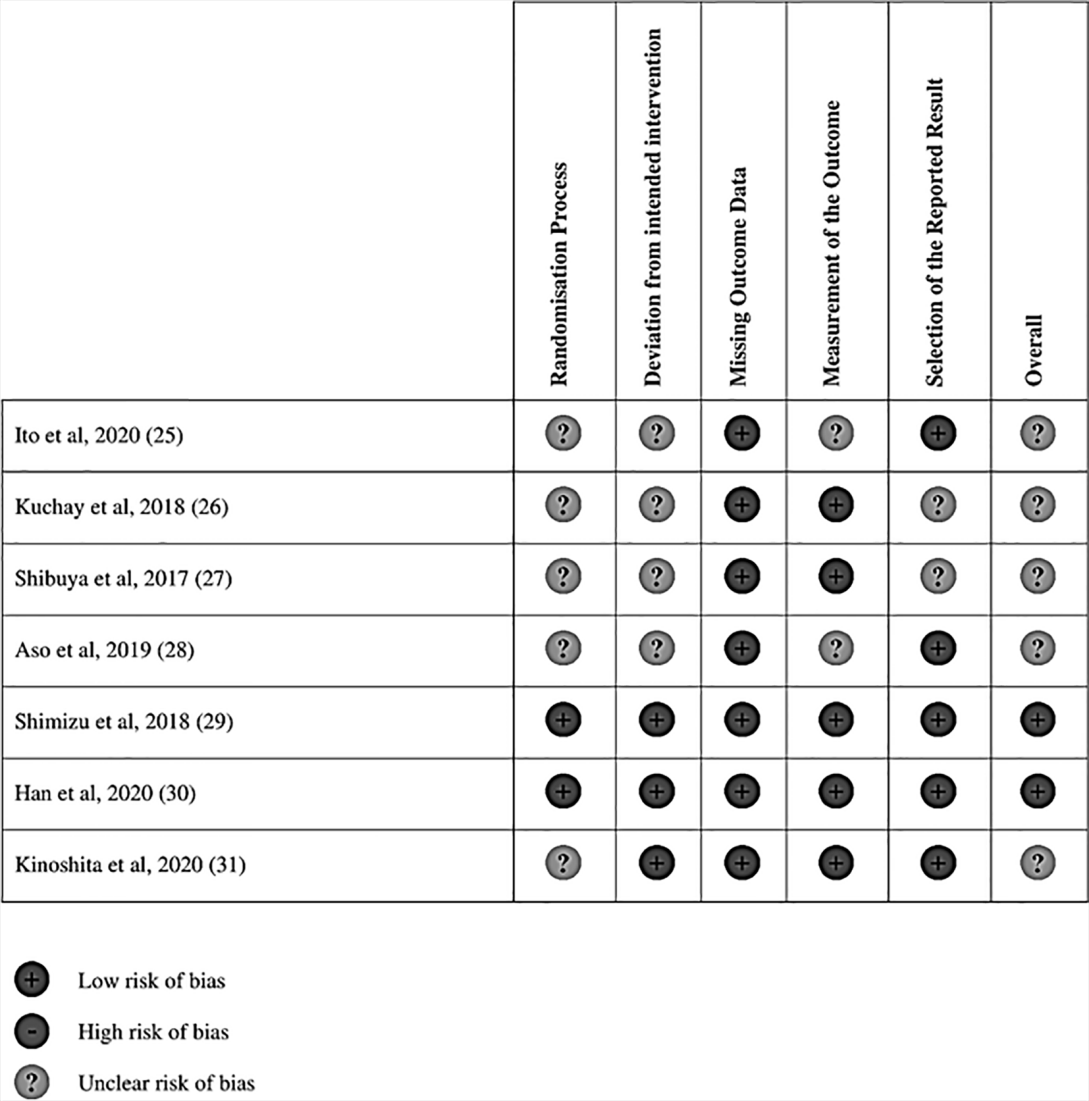
Risk of Bias Assessment for RCTs.

Electronic database searches identified 1,372 articles after duplicate removal. 93 articles were selected for full text review, of which 10 met the inclusion criteria, inclusive of 7 randomized controlled trials (RCT) (25–31). **Figure 1** shows the flowchart of the review. Of the 10 articles, one compared SGLT2i to thiazolidinediones (TZD) (25), two compared SGLT2i to incretin-based therapies (32, 33), two compared SGLT2i to metformin (27), five compared SGLT2i to non-SGLT2i therapies (26, 28–30, 34), and one was a three-arm study which compared SGLT2i, TZD and insulin-based therapies (31). In total, there were 555 patients, with 260 on SGLT2i, 67 on TZD, 73 on incretin-based therapies, 33 on insulin-based therapies, 16 on metformin and the remaining 106 on non-SGLT2i treatments. The SGLT2i treatments consist of Canagliflozin, Dapagliflozin, Empagliflozin, Ipragliflozin and Luseogliflozin. All patients were clinically diagnosed with type 2 diabetes and NAFLD, with two studies reporting patients with NASH (n = 96), fibrosis (n = 78) and cirrhosis (n = 12) based on liver biopsy. The duration of treatment for patients in included studies ranged from twenty-four weeks to more than three years. The glycosylated hemoglobin (HbA1c) ranged from 6.0% to 12.0%. The characteristics of patients in included papers are presented in **Table 1**. Quality assessment of included articles are found in **Supplementary Material 8** and **Figure 2**.

**Table 1:** Summary of Included Articles.

Author, year	Country	Studydesign, yrial number	Comparison	SGLT2 (n)	Control (n)	Male (%)		Age		Weight (kg)		BMI (kg/m^2^)		HbA1c (%)		NASH (n)	Fibrosis (n)	Cirrhosis (n)	Diagnosis Method
						SGLT2	Control	SGLT2	Control	SGLT2	Control	SGLT2	Control	SGLT2	Control				
Ito et al. (25)	Japan	RCT (UMIN000022651)	SGLT2 (Ipragliflozin) vs. TZD	32	34	44	53	57.3 ± 12.1	59.1 ± 9.80	79.6 ± 17.9	76.7 ± 15.2	30.7 ± 5.0	29.9 ± 6.2	8.5 ± 1.5	8.3 ± 1.4	–	–	–	CT or Ultrasound
Seko et al. (32)	Japan	Retrospective Study	SGLT2 (Canagliflozin & Ipragliflozin) vs. Incretin-based therapy	24	21	37.5	38.1	60.3 ± 8.82	59.4 ± 17.0	–	–	29.6 ± 3.43	29.2 ± 6.87	6.7 ± 0.49	7.0 ± 1.37	38	33	4	Biopsy
Choi et al. (33)	Korea	Retrospective Study	SGLT2 (Dapagliflozin) vs. Incretin-based therapy	50	52	62	65.4	50.7 ± 10.2	56.6 ± 7.4	79.8 ± 14.6	73.7 ± 12.8	–	–	8.3 ± 1.7	7.4 ± 1.5	–	–	–	Ultrasound
Kuchay et al. (26)	India	RCT (NCT02686476)	SGLT2 (Empagliflozin) vs. No SGLT2	22	20	–	–	–	–	80.8 ± 13.0	81.1 ± 16.1	30.0 ± 3.80	29.4 ± 3.1	9.0 ± 1.0	9.1 ± 1.4	–	–	–	MRI-PDFF
Shibuya et al.^a^ (27)	Japan	RCT (UMIN000016090)	SGLT2 (Luseogliflozin) vs. Metformin	16	16	62.5	50	53.3 ± 11.1	59.7 ± 9.63	76.3 ± 16.6	75.4 ± 17.4	27.6 ± 1.85	28.0 ± 5.26	7.63 ± 0.52	7.3 ± 0.59	–	–	–	CT or Ultrasound
Aso et al.^a^ (28)	Japan	RCT (UMIN000022155)	SGLT2 (Dapagliflozin) vs. No SGLT2	33	24	–	–	–	–	72.1 ± 14.0	74.0 ± 12.3	27.6 ± 4.7	28.7 ± 3.5	8.37 ± 1.48	7.70 ± 1.24	–	–	–	Ultrasound
Shimizu et al.^a^ (29)	Japan	RCT (UMIN000022155)	SGLT2 (Dapagliflozin) vs. No SGLT2	33	24	57.6	62.5	56.2 ± 11.5	57.1 ± 13.8	73.9 ± 16.1	76.4 ± 13.9	27.6 ± 4.70	28.3 ± 3.5	8.37 ± 1.48	7.70 ± 1.24	–	–	–	Ultrasound
Han et al. (30)	Korea	RCT (NCT02875821)	SGLT2 (Ipragliflozin) vs. No SGLT2	29	15	63.3	60	52.5 ± 10.3	56.7 ± 11.8	84.3 ± 17.2	81.4 ± 8.5	30.6 ± 5.30	30.2 ± 2.5	6.7 ± 0.7	6.6 ± 0.6	–	–	–	Ultrasound
Yano et al. (34)	Japan	Retrospective study	SGLT2 (Dapagliflozin, Canagliflozin, Ipragliflozin, Empagliflozin) vs. No SGLT2	22	47	40.9	50	57.8 ± 9.25	59.0 ± 9.50	–	–	30.5 ± 3.55	26.2 ± 5.28	7.55 ± 1.2	6.90 ± 1.38	58	45	8	Biopsy
Kinoshita et al. (31)	Japan	RCT (UMIN000021291)	SGLT2 (Dapagliflozin) vs. Insulin-based therapy	32	33	46.9	45.5	58.7 ± 9.05	58.0 ± 13.2	77.1 ± 16.4	75.7 ± 15.5	29.5 ± 4.53	28.4 ± 4.02	7.38 ± 0.91	7.57 ± 0.86	–	–	–	L/S Ratio, CT Scan
Kinoshita et al. (31)	Japan	RCT (UMIN000021291)	SGLT2 (Dapagliflozin) vs. TZD	32	33	46.9	45.5	58.7 ± 9.05	59.0 ± 10.9	77.1 ± 16.4	75.0 ± 19.0	29.5 ± 4.53	28.7 ± 5.17	7.38 ± 0.91	7.39 ± 1.03	–	–	–	L/S Ratio, CT Scan

^a^The two papers were based on the same study.

### Changes in Body Composition

As expected, SGLT2i treatment reduced body weight, waist circumference, subcutaneous adipose tissue (SAT) and visceral adipose tissue (VAT). The reduction in body weight was significantly greater compared to controls (SMD: -2.317, CI: -3.576 to -1.057, p < 0.001), TZD (SMD: -4.817, CI: -9.201 to -0.433, p = 0.031), incretin-based therapies (SMD: -0.589, CI: -0.986 to -0.192, p = 0.004) and insulin-based therapies (SMD: -2.074, CI: -2.681 to -1.468, p < 0.001) (see **Supplementary Material 2**). The reduction in BMI was significantly greater when compared to controls (SMD: -1.092, CI: -2.032 to -0.153, p = 0.023) and metformin (SMD: -1.120, CI: -1.869 to -0.371, p = 0.003). The reduction of VAT was statistically significant after SGLT2i treatment (SMD: -0.277, CI: -0.511 to -0.043, p = 0.02) and was greater when compared to controls (SMD: -2.247, CI: -3.586 to -0.907, p = 0.001), insulin-based therapies (SMD: -1.179, CI: -1.707 to -0.651, p < 0.001) and metformin (SMD: -1.145, CI: -1.896 to -0.394, p = 0.003). The reduction in SAT reached statistical significance when compared against TZD (SMD: -6.347, CI: -7.547 to -5.146, p < 0.001).

### Changes in Metabolic Parameters

SGLT2i treatment significantly decreased fasting glucose (SMD: -0.326, CI: -0.634 to -0.017, p = 0.039), HbA1c (SMD: -0.701, CI: -1.098 to -0.303, p = 0.001), triglyceride levels (SMD: -0.230, CI: -0.409 to -0.052, p = 0.011) (see **Supplementary Material 3**).

**Figure 3:**
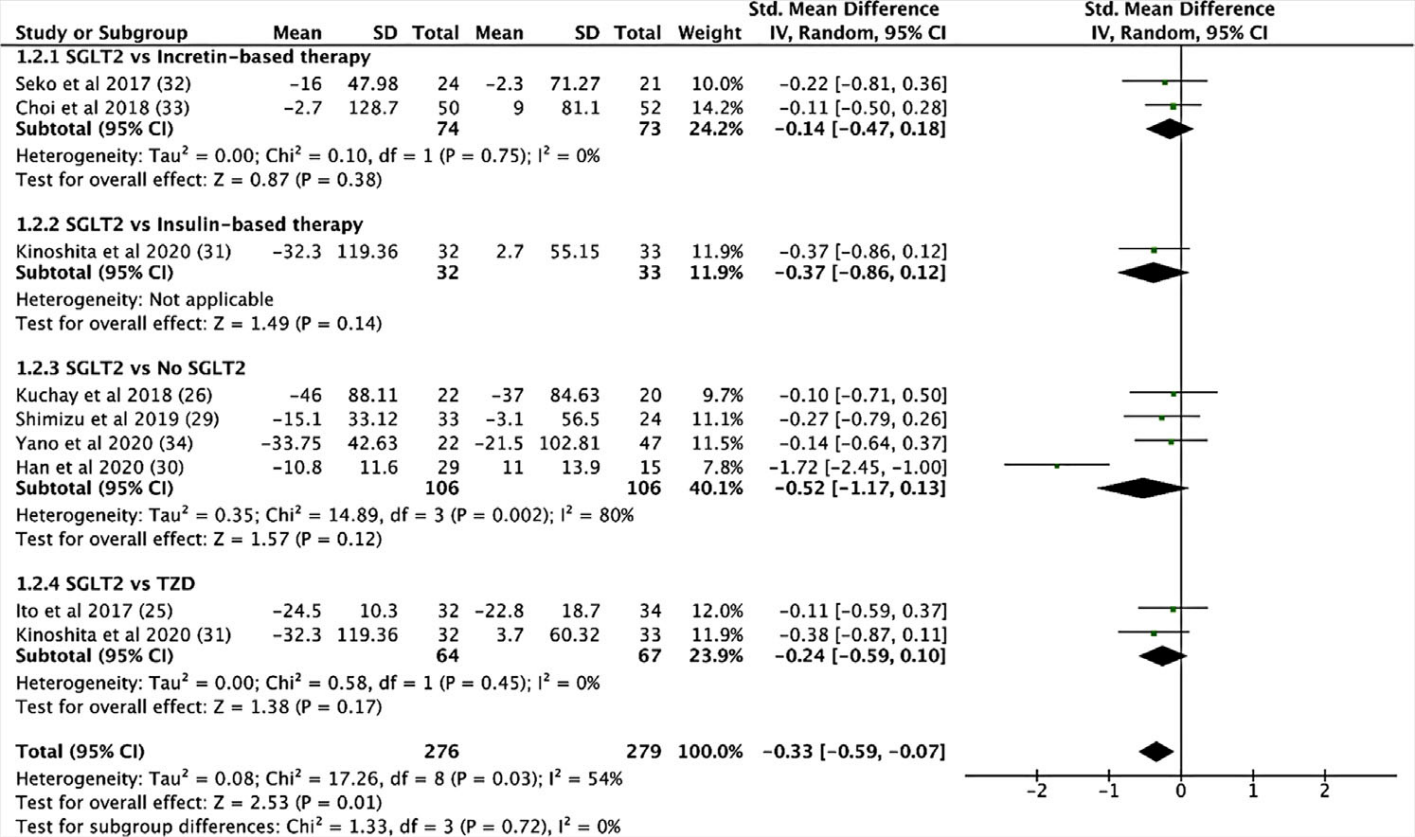
SGLT2 vs. Control, Triglycerides Levels.

Compared to controls, SGLT2i treatment resulted in a significantly lower triglyceride levels (SMD: -0.336, CI: -0.597 to -0.076, p = 0.011, **Figure 3**). Compared to TZD, SGLT2i treatment had a greater reduction in total cholesterol levels (SMD: -1.545, CI: -2.096 to -0.993, p < 0.001). Compared to incretin-based therapy, SGLT2i treatment showed a greater reduction in fasting glucose (SMD: -0.841, CI: -1.321 to -0.360, p = 0.001). Compared to insulin-based therapies, SGLT2i treatment resulted in a significant higher HDL levels (SMD: 0.861, CI: 0.352 to 1.370, p = 0.001). Compared to metformin, SGLT2i treatment led to a greater reduction in HbA1c levels (SMD: -0.825, CI: -1.548 to -0.101, p = 0.026). The comparisons between SGLT2i treatment with other glucose-lowering agents on the indices of insulin resistance (fasting insulin, HOMA-IR, CPR, adipo-IR), beta-cell function (CPR index, HOMA-B), systolic and diastolic blood pressure (SBP), diastolic blood pressure (DBP), non-esterified fatty acids (NEFA) and low-density lipoprotein (LDL) levels were non-significant.

### Changes in Adipokines and Inflammatory Markers

SGLT2i treatment resulted in an increase in adiponectin levels (SMD: 0.301, CI: 0.005 to 0.596, p = 0.046) (see **Supplementary Material 4**). There were no differences in the change in adiponectin levels for the comparison between SGLT2i treatment and TZD or insulin-based therapies. SGLT2i treatment significantly reduced soluble dipeptidyl peptidase 4 (sDPP-4) levels (SMD: -0.764, CI: -1.264 to -0.264, p = 0.003) and the reduction was greater when compared to controls (SMD: -0.638, CI: -1.177 to -0.099, p = 0.02).

### Changes in Steatosis Markers

SGLT2i treatment significantly reduced hepatic fat content, as measured by the magnetic resonance imaging proton density fat fraction (MRI-PDFF) (SMD: -0.789, CI: -1.404 to -0.175, p = 0.012) and when compared to control (SMD: -0.923, CI:-1.562 to -0.285, p = 0.005). SGLT2i treatment significantly improved the liver-to-spleen (L/S) attenuation ratios as measured by computed tomography (CT) scans (SMD: 0.456, CI: 0.142 to 0.771, p = 0.004) (see **Supplementary Material 5**), and the improvement was greater compared to insulin-based therapies (SMD: 0.614, CI: 0.116 to 1.112, p = 0.016) or metformin (SMD: 1.957, CI: 1.105 to 2.809, p < 0.001). SGLT2i treatment had a greater reduction in CAP scores (SMD: -1.376, CI: -2.540 to -0.213, p = 0.02) when compared to controls. The hepatic steatosis index (HSI) was lower after SGLT2i treatment but did not reach statistical significance.

### Changes in Fibrosis Markers

SGLT2i treatment also reduced the numerical measured fibrosis markers such as FIB-4 index, liver stiffness measurement (by transient elastography), Mac-2 Binding Protein but did not reach statistical significance (see **Supplementary Material 6**). There was no significant change in the NAFLD fibrosis score but significantly reduced NAFIC score (predicting NASH) (SMD: -0.569, CI: -1.062 to -0.077, p = 0.023) and serum ferritin levels (SMD: -0.409, CI: -0.694 to -0.124, p = 0.005) was found. Similarly, when compared to controls, SGLT2i treatment resulted in significant reductions in all the fibrosis markers but only reached statistical significance for NAFIC score (SMD: -0.692, CI: -1.233 to -0.15), p = 0.012). Compared to TZD, SGLT2i treatment resulted in a significantly lower FIB-4 index values (SMD: -0.780, CI: -1.281 to -0.279, p = 0.002).

### Changes in Liver and Renal Biomarkers

**Figure 4:**
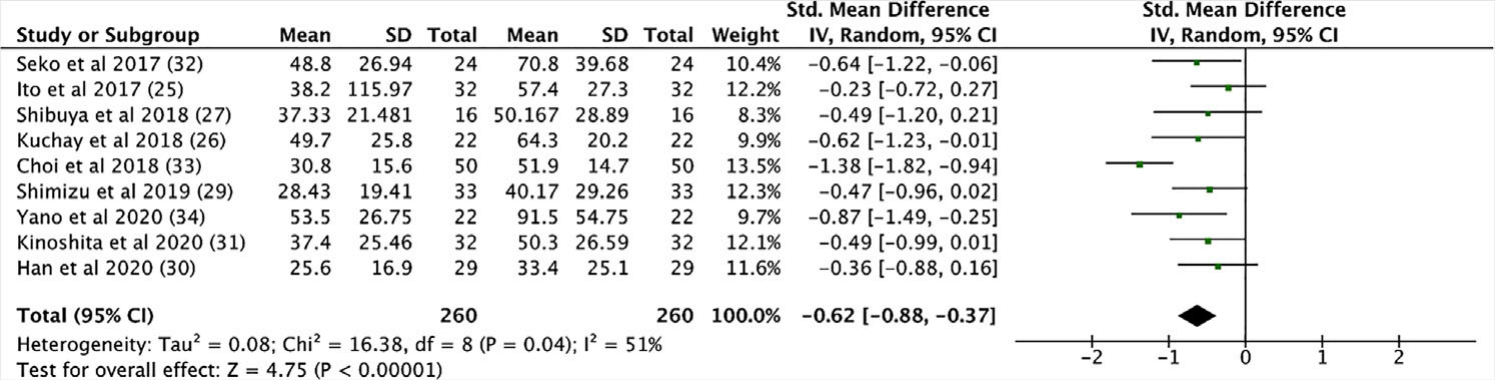
Before and After Comparison of ALT Levels.

**Figure 5:**
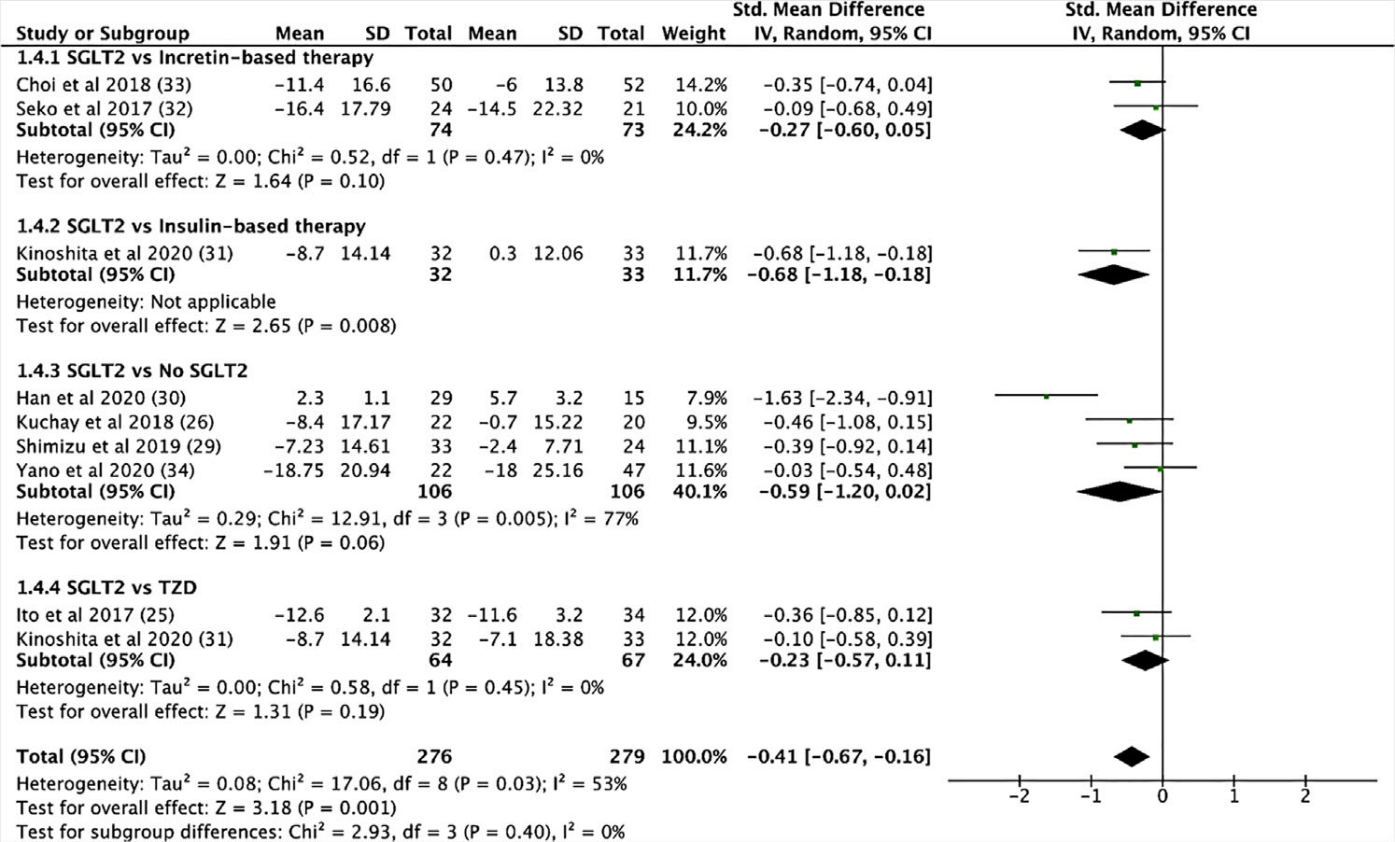
SGLT2i vs. Control, AST Levels.

SGLT2i treatment significantly reduced aspartate aminotransferase (AST) levels (SMD: -0.539, CI: -0.720 to -0.357, p < 0.001), alanine transaminase (ALT) levels (SMD: -0.633, CI: -0.892 to -0.373, p < 0.001, **Figure 4**), and gamma-glutamyl transferase (GGT) levels (SMD: -0.330, CI: -0.530 to -0.129, p = 0.001) (see **Supplementary Material 7**). In addition, SGLT2i treatment also resulted in significant increases in albumin levels (SMD: 0.353, CI: 0.034 to 0.671, p = 0.03). Compared to controls, SGLT2i resulted in a significant decrease in ALT levels (SMD: -0.468, CI: -0.685 to -0.251, p < 0.001) and AST levels (SMD: -0.421, CI: -0.680 to -0.161, p = 0.001, **Figure 5**) and increase in albumin levels (SMD: 0.363, CI: 0.047 to 0.678, p = 0.024). Compared to insulin-based therapies, SGLT2i treatment had a significantly lower AST levels (SMD: -0.686, CI: -1.186 to -0.185, p = 0.007), ALT levels (SMD: -0.551, CI: -1.046 to -0.055, p = 0.029) and GGT levels (SMD: -0.639, CI: -1.138 to -0.140, p = 0.012). Total bilirubin, platelet count, uric acid levels and eGFR were largely unchanged.

## Discussion

To the best of our knowledge, this is a comprehensive meta-analysis that analyses the effectiveness of SGLT2i on the improvement of NAFLD and hepatic fibrosis specific to type 2 diabetes population. While there are currently no established guidelines on the pharmacological treatment of NAFLD in type 2 diabetic patients (2), pioglitazone has been shown to improve the liver histology among pre-diabetics, diabetics and non-diabetics with NAFLD and NASH (35, 36). However, pioglitazone has some serious side effects including weight gain, fluid retention, hospitalization of heart failure, and bone loss (2). More recently, there have been reports on another oral glucose-lowering agent, SGLT2i, in improving NAFLD (37–39). In our analysis, SGLT2i was shown to result in significant improvement in hepatic steatosis, body composition, metabolic parameters and liver biomarkers, which indicates promise of SGLT2i as a treatment modality in patients with concurrent NAFLD and type 2 diabetes mellitus.

Unlike chronic kidney disease, where serum creatinine, urinary albumin-to-creatinine ratio and estimated glomerular filtration rate could be used to identify and as biomarkers for therapeutic efficacy, such biomarkers for NAFLD are less known. Liver biopsies are the gold standard to diagnose and prognosticate NAFLD and NASH (40), but are associated with significant risks to the patients (41). Thus, surrogate indicators such as MRI-PDFF, L/S ratios, CAP scores and HSI values have been used to quantify the degree of hepatic steatosis (42). Non-invasive markers of liver fibrosis such as FIB-4 index, NFS and TEM (42), and serum biomarkers such as ALT, AST and GGT have been associated with NAFLD and NASH (43, 44).

In this analysis, SGLT2i treatment was associated with improvement in hepatic steatosis as measured by MRI-PDFF, L/S attenuation ratios, CAP score and HSI values. In the animal studies, SGLT2i has been shown to decrease *de novo* lipogenesis and increase lipolysis, and this has been postulated as the mechanism on how SGLT2i improves hepatic steatosis (45). Studies have shown that SGLT2i results in elevation of glucagon and a change in the insulin-to-glucagon ratio, which favours lipolysis and ketogenesis in the liver (46). Metformin is a very potent oral glucose-lowering agent and can reduce hepatic glucose production and improve insulin sensitivity (47). In a meta-analysis, metformin improves liver function, HOMA-IR and BMI to some extent, but not histological response in NAFLD patients (48). Our results showed that SGLT2i had greater effectiveness in reducing hepatic steatosis compared to metformin. Insulin is an anabolic hormone that results in weight gain and increased lipogenesis (49). Juurinen L et al. showed that chronic insulin therapy for type 2 diabetic patients resulted in weight gain but a slight significant reduction in liver fat content with improved hepatic liver sensitivity (50). Compared with insulin-based therapies, SGLT2i had a greater effect on reducing hepatic fat content, in addition to that of significant weight loss and reduced visceral adipose tissue (51).

NAFIC score is a non-invasive scoring system for predicting NASH in Japanese NAFLD and is a derivative of serum ferritin, fasting insulin and plasma type IV collagen 7S concentrations (52). This study showed that SGLT2i significantly improved the NAFIC score and serum ferritin. It might indicate that serum ferritin could be a biomarker of choice to inform physicians on the therapeutic efficacy in improving NASH and liver fibrosis. The effect of SGLT2i on other known indicators of liver fibrosis such as in FIB-4 index and NFS, and both liver fibrosis and cirrhosis for liver stiffness measurements were largely non-significant. The stage of NAFLD (i.e. fatty infiltration, inflammation or fibrosis) or the duration of treatment might have influenced the therapeutic efficacy of SGLT2i on the biomarkers and other non-invasive measurements in this meta-analysis.

Pioglitazone has shown benefits in liver function, liver fat, and NASH resolution (35). In this meta-analysis, SGLT2i treatment had a larger decrease in FIB-4 index values compared to TZD-based therapy. Similar to the comparison with insulin-based therapy, the SGLT2i treatment showed a reduction in body weight and SAT compared to TZD-based therapy. Whether weight loss attributed to SGLT2i added advantage over TZD-based therapy is currently not known. The American Association for the Study of Liver Diseases (AASLD) recommended pharmacologic treatment such as pioglitazone in patients with biopsy-proven NASH and fibrosis, since patients without fibrosis generally have a favorable prognosis (2). Whether this recommendation will extend to SGLT2i awaits the outcomes from ongoing RCT on effectiveness of SGLT2i treatment on biopsy-proven NASH (53).

Consistent with the improvement in the NAFIC score, serum ferritin and steatosis biomarkers, there were significant improvements in the ALT and AST levels after SGLT2i treatments and when compared to controls or insulin-based therapy. While the accuracy of raised ALT and AST as biomarkers of NASH is low (54), they are commonly utilized as clinical indicators of hepatocellular injury and improvement in these levels indicates improvement in fatty liver or NASH (55).

SGLT2i treatment significantly reduced body weight and BMI, and improved body fat composition (SAT and VAT), which are consistent with current diabetic studies (56). Weight loss and reduction in VAT is strongly correlated with the decrease in hepatic fat (57), and is a key factor in the improvement in liver histology in NASH patients (58). Improvement in the body fat composition is also associated with an increase in adiponectin levels, an adipokine that is associated with improved insulin sensitivity (59). In our analysis, SGLT2i also resulted in improved insulin sensitivity, as measured by HOMA-IR, but did not reach statistical significance. While both Shimuzu et al. and Han et al. showed a significant decrease in the HOMA-IR after SGLT2i use (28, 30), the baseline values of the patients included in the two studies were lower compared to studies which had non-significant changes in HOMA-IR values (25, 31, 32). This could potentially indicate that the effects of SGLT2i is stronger in patients with higher baseline insulin sensitivity. However, further studies are required to determine the efficacy of SGLT2i on patients with differing insulin sensitivity levels. It is widely recognized that SGLT2i confer cardiorenal benefits among people with and without type 2 diabetes (60), but it is currently not clear whether the benefits of SGLT2i on cardiorenal protection will extend to NAFLD with and without type 2 diabetes.

### Limitations

There are several limitations in this meta-analysis. The studies included in this meta-analysis were mainly from Asian countries, which resulted in less representation from the West. However, NAFLD is a common disease with general underlying pathophysiology of insulin resistance and metabolic syndrome and thus, we believe that the results of this study could be generalized to other regions in the world. In addition, we were only able to include 7 RCTs in this paper due to the limited number of clinical trials available, with 5 out of 7 RCTs deemed to have an unclear risk of bias. This indicates strong possibility of bias in the results of this analysis, and thus prompts the need for more clinical trials to better examine the efficacy of SGLT2i, especially vis-à-vis other anti-diabetic medication, on the improvement of NAFLD in patients with concomitant type 2 diabetes. The focus of this meta-analysis was on patients with concomitant NAFLD and type 2 diabetes, but we believe the effect of SGLT2i could be extended to patients with NAFLD without type 2 diabetes as the effect of SGLT2i on improving NAFLD is independent of glycaemia, similar to the cardiorenal protection. Due to the invasive nature of liver biopsy, many of the included studies did not report liver biopsy as a diagnostic tool but relied on ultrasonography, CT scans and MRI to diagnose NAFLD, which are less accurate in demonstrating improvements in NASH (61, 62). SGLT2i treatments have been known to result in a mean weight loss of 3% (63). In the LookAhead study (NCT00017953), a weight loss between 1 and 5% was associated with a mean 33.3% improvement in hepatic steatosis (64). Thus, we are not able to differentiate the direct effect to SGLT2i and the indirect effect of weight loss from SGLT2i on the improvement in NAFLD.

## Conclusion

In conclusion, SGLT2i treatment improves hepatic steatosis, body composition and adiponectin levels and to some extent liver fibrosis. SGLT2i could be considered as a potential treatment strategy among type 2 diabetic patients with NAFLD after further discussions with physicians, if there are no contraindications. More studies are needed to understand the direct and indirect effects of SGLT2i on the prognosis and mortality from NAFLD.

## Data Availability Statement

The original contributions presented in the study are included in the article/**Supplementary Material**. Further inquiries can be directed to the corresponding author.

## Author Contributions

CW: Literature Search, data extraction, data analysis and interpretation, drafting and critical revisions of the manuscript. CY: Literature search, data extraction, and data analysis and interpretation. CN: Study conception, literature search, methodology, data analysis and interpretation, critical revision of article and final approval. YC: Data analysis and interpretation, critical revision of article and final approval. YL: Drafting of article and critical revision of article. AL: Critical revision of the article and final approval. MM: Data interpretation, critical revision of the article and final approval. CK: Study conception, methodology, data interpretation, critical revision of article and final approval. All authors contributed to the article and approved the submitted version.

## Conflict of Interest

The authors declare that the research was conducted in the absence of any commercial or financial relationships that could be construed as a potential conflict of interest.

The reviewer KV declared a shared affiliation with the authors to the handling editor at time of review.
