# Supplementary material for: Sodium-Glucose Co-Transporter 2 Inhibitors for Non-Alcoholic Fatty Liver Disease in Asian Patients With Type 2 Diabetes: A Meta-Analysis
Source: Front Endocrinol (Lausanne). 2021 Feb 11;11:609135. doi: 10.3389/fendo.2020.609135 (PMC7905212; doi:10.3389/fendo.2020.609135)
Supplement: Supplementary file 1 [file Table_1.docx]

**Sodium Glucose Co-transporter 2** **Inhibitors for Non-alcoholic Fatty Liver Disease in Type 2 Diabetes: A Meta-analysis**

Chloe Wong^ψ1^, Clyve Yu Leon Yaow^ψ1^, Cheng Han Ng^1^, Yip Han Chin^1^, Yi Fen Low^1^, Amanda Yuan Ling Lim^1,2^, Mark Dhinesh Muthiah^1,2,3^, Chin Meng Khoo*^1,2^

^1^Yong Loo Lin School of Medicine, National University of Singapore, Singapore,

^2^Department of Medicine, National University Hospital, Singapore

^3^National University Centre for Organ Transplantation, National University Hospital, Singapore

^ψ^The authors had equal contribution

***Correspondence:**

*Chin Meng Khoo*

Yong Loo Lin School of Medicine,

10 Medical Dr, Singapore 117597

Email: [mdckcm@nus.edu.sg](mailto:mdckcm@nus.edu.sg)

**Supplementary Material 1**. MEDLINE Search Strategy

| **#1** | exp Non-alcoholic Fatty Liver Disease/ or NAFLD.tw. or Hepatic Fat.tw. or ((liver or hepat*) adj3 (fatty or steatosis or steatoses)).tw. |
| --- | --- |
| **#2** | exp Fatty Liver/ or Non Alcoholic SteatoHepatitis.tw. or Non Alcoholic Steato hepatitis.tw. or ((fatty adj3 (liver* or hepat*)) or steatohepat* or NAFL* or NASH*).tw. |
| **#3** | exp Sodium-Glucose Transporter 2/ or exp pioglitazone/ or gliflozin?.tw. or ((SGLT2 or SGLT 2) adj2 inhibitor?).tw. or (sodium adj2 glucose adj (cotransporter or co transporter) adj2 inhibitor?).tw. or (atigliflozin or bexagliflozin or canagliflozin or dapagliflozin or empagliflozin or ertugliflozin or ipragliflozin or licogliflozin or luseogliflozin or remogliflozin or sergliflozin or sotagliflozin or tofogliflozin or pioglitazone).tw. |
| **#4** | (animals not humans).sh. |
| **#5** | #1 OR #2 |
| **#6** | #3 AND #5 |
| **#7** | #6 NOT #4 |

**Supplementary Material 2:** Changes in Body Composition

|  | **After SGLT2i Treatment** | | **Comparisons against Controls** | | **Comparisons against TZD** | | **Comparisons against Incretin-based Therapies** | | **Comparisons against Insulin-based Therapies** | | **Comparisons against Metformin** | |
| --- | --- | --- | --- | --- | --- | --- | --- | --- | --- | --- | --- | --- |
|  | **SMD (95% CI)** | **P-value** | **SMD (95% CI)** | **P-value** | **SMD (95% CI)** | **P-value** | **SMD (95% CI)** | **P-value** | **SMD (95% CI)** | **P-value** | **SMD (95% CI)** | **P-value** |
| Body Weight | -0.154 (-0.351 to 0.043) | 0.126 | -2.317 (-3.576 to -1.057) | **<0.001** | -4.817 (-9.201 to -0.433) | **0.031** | -0.589 (-0.986 to -0.192) | **0.004** | -2.074 (-2.681 to -1.468) | **<0.001** | NA | NA |
| BMI | -0.225 (-0.456 to 0.005) | 0.055 | -1.092 (-2.032 to -0.153) | **0.023** | NA | NA | -0.304 (-0.893 to 0.285) | 0.312 | NA | NA | -1.120 (-1.869 to -0.371) | **0.003** |
| Waist Circumference | -0.268 (-0.785 to 0.249) | 0.31 | -4.817 (-6.021 to -3.613) | **<0.001** | NA | NA | NA | NA | NA | NA | NA | NA |
| Subcutaneous Adipose Tissue | -0.094 (-0.380 to 0.192) | 0.52 | -2.534 (-5.299 to 0.231) | 0.072 | -6.347 (-7.547 to -5.146) | **<0.001** | NA | NA | NA | NA | NA | NA |
| Visceral Adipose Tissue | -0.277 (-0.511 to -0.043) | **0.02** | -2.247 (-3.586 to -0.907) | **0.001** | -2.797 (-6.672 to 1.078) | 0.157 | NA | NA | -1.179 (-1.707 to -0.651) | **<0.001** | -1.145 (-1.896 to -0.394 | **0.003** |

Bolded outcome is statistically significant, p<0.05

**Supplementary Material 3:** Changes in Metabolic Parameters

|  | **After SGLT2i Treatment** | | **Comparisons against Controls** | | **Comparisons against TZD** | | **Comparisons against Incretin-based Therapies** | | **Comparisons against Insulin-based Therapies** | | **Comparisons against Metformin** | |
| --- | --- | --- | --- | --- | --- | --- | --- | --- | --- | --- | --- | --- |
|  | **SMD (95% CI)** | **P-value** | **SMD (95% CI)** | **P-value** | **SMD (95% CI)** | **P-value** | **SMD (95% CI)** | **P-value** | **SMD (95% CI)** | **P-value** | **SMD (95% CI)** | **P-value** |
| Fasting Glucose | -0.326 (-0.634 to -0.017) | **0.039** | -0.191 (-0.529 to 0.147) | 0.268 | 0.123 (-0.406 to 0.651) | 0.65 | -0.841 (-1.321 to -0.360) | **0.001** | -0.318 (-0.808 to 0.171) | 0.202 | 0.224 (-0.471 to 0.920) | 0.527 |
| HbA1c | -0.701 (-1.098 to -0.303) | **0.001** | -0.210 (-0.603 to 0.183) | 0.295 | 0.422 (-0.500 to 1.344) | 0.37 | -0.252 (-0.577 to 0.073) | 0.129 | -0.251 (-0.739 to 0.237) | 0.314 | -0.825 (-1.548 to -0.101) | **0.026** |
| Fasting Insulin | 0.033 (-0.220 to 0.286) | 0.797 | 0.195 (-0.298 to 0.688) | 0.439 | 0.628 (-0.243 to 1.499) | 0.157 | 0.280 (-0.308 to 0.869) | 0.351 | -0.217 (-0.705 to 0.271) | 0.383 | NA | NA |
| C-Peptide Immunoreactivity | 0.160 (-0.535 to 0.854) | 0.652 | 0.209 (-0.486 to 0.904) | 0.555 | NA | NA | NA | NA | NA | NA | 0.209 (-0.486 to 0.904) | 0.555 |
| C-Peptide Immunoreactivity Index | 0.816 (0.093 to 1.539) | **0.027** | -0.063 (-0.757 to 0.630) | 0.858 | NA | NA | NA | NA | NA | NA | -0.063 (-0.757 to 0.630) | 0.858 |
| HOMA-IR | -0.129 (-0.396 to 0.139) | 0.346 | -0.132 (-0.750 to 0.486) | 0.676 | 0.575 (-0.560 to 1.711) | 0.321 | 0.184 (-0.403 to 0.771) | 0.539 | -0.336 (-0.826 to 0.154) | 0.179 | NA | NA |
| HOMA-beta | -0.214 (-0.730 to 0.302) | 0.416 | -2.207 (-2.989 to -1.425) | **<0.001** | NA | NA | NA | NA | NA | NA | NA | NA |
| Adipo-IR | 0.021 (-0.469 to 0.511) | 0.934 | 2.002 (1.408 to 2.597) | **<0.001** | 2.002 (1.408 to 2.597) | **<0.001** | NA | NA | NA | NA | NA | NA |
| Total Cholesterol | -0.196 (-0.418 to 0.026) | 0.084 | -0.079 (-1.000 to 0.842) | 0.866 | -1.545 (-2.096 to -0.993) | **<0.001** | -0.152 (-0.476 to 0.172) | 0.358 | NA | NA | NA | NA |
| Triglycerides | -0.230 (-0.409 to -0.052) | **0.011** | -0.336 (-0.597 to -0.076) | **0.011** | -0.245 (-0.598 to 0.099) | 0.163 | -0.146 (-0.470 to 0.179) | 0.379 | -0.378 (-0.869 to 0.112) | 0.131 | NA | NA |
| HDL | 0.144 (-0.034 to 0.322) | 0.113 | 0.302 (-0.042 to 0.646) | 0.085 | 0.198 (-0.146 to 0.542) | 0.259 | -0.129 (-0.454 to 0.195) | 0.435 | 0.861 (0.352 to 1.370) | **0.001** | NA | NA |
| LDL | -0.165 (-0.343 to 0.014) | 0.07 | -0.139 (-0.834 to 0.557) | 0.696 | -1.400 (-4.006 to 1.207) | 0.293 | -0.307 (-0.798 to 0.184) | 0.221 | -0.168 (-0.655 to 0.319) | 0.498 | NA | NA |
| Systolic Blood Pressure | -0.092 (-0.351 to 0.167) | 0.485 | -0.524 (-1.230 to 0.182) | 0.146 | -0.620 (-1.481 to 0.241) | 0.158 | NA | NA | -0.231 (-0.719 to 0.257) | 0.354 | NA | NA |
| Diastolic Blood Pressure | 0.095 (-0.164 to 0.354) | 0.473 | -0.067 (-0.303 to 0.170) | 0.58 | -0.07 (-0.566 to 0.426) | 0.782 | NA | NA | -0.123 (-0.610 to 0.363) | 0.62 | NA | NA |
| NEFA | -0.285 (-0.921 to 0.351) | 0.38 | 0.607 (-0.497 to 1.712) | 0.281 | 0.902 (-0.878 to 2.683) | 0.32 | NA | NA | 0.035 (-0.452 to 0.521) | 0.889 | NA | NA |

Bolded outcome is statistically significant, p<0.05

**Supplementary Material 4:** Changes in Adipokines and Inflammatory Markers

|  | **After SGLT2i Treatment** | | **Comparisons against Controls** | | **Comparisons against TZD** | | **Comparisons against Incretin-based Therapies** | | **Comparisons against Insulin-based Therapies** | | **Comparisons against Metformin** | |
| --- | --- | --- | --- | --- | --- | --- | --- | --- | --- | --- | --- | --- |
|  | **SMD (95% CI)** | **P-value** | **SMD (95% CI)** | **P-value** | **SMD (95% CI)** | **P-value** | **SMD (95% CI)** | **P-value** | **SMD (95% CI)** | **P-value** | **SMD (95% CI)** | **P-value** |
| Adiponectin | 0.301 (0.005 to 0.596) | **0.046** | -1.683 (-3.505 to 0.139) | 0.07 | -3.646 (-8.060 to 0.768) | 0.105 | NA | NA | 0.081 (-0.406 to 0.567) | 0.745 | NA | NA |
| Leptin | 0.041 (-0.442 to 0.523) | 0.869 | -0.102 (-0.628 to 0.424) | 0.705 | NA | NA | NA | NA | NA | NA | NA | NA |
| sDPP-4 | -0.764 (-1.264 to -0.264) | **0.003** | -0.638 (-1.177 to -0.099) | **0.02** | NA | NA | NA | NA | NA | NA | NA | NA |
| TGF-beta 1 | -0.461 (-0.950 to 0.028) | 0.065 | -0.326 (-0.855 to 0.203) | 0.227 | NA | NA | NA | NA | NA | NA | NA | NA |

*Bolded outcome is statistically significant, p<0.05

**Supplementary Material 5:** Changes in Steatosis Markers

|  | **After SGLT2i Treatment** | | **Comparisons against Controls** | | **Comparisons against TZD** | | **Comparisons against Incretin-based Therapies** | | **Comparisons against Insulin-based Therapies** | | **Comparisons against Metformin** | |
| --- | --- | --- | --- | --- | --- | --- | --- | --- | --- | --- | --- | --- |
|  | **SMD (95% CI)** | **P-value** | **SMD (95% CI)** | **P-value** | **SMD (95% CI)** | **P-value** | **SMD (95% CI)** | **P-value** | **SMD (95% CI)** | **P-value** | **SMD (95% CI)** | **P-value** |
| L/S Ratio | 0.456 (0.142 to 0.771) | **0.004** | 0.590 (-0.124 to 1.305) | 0.106 | 0.033 (-0.460 to 0.526) | 0.895 | NA | NA | 0.614 (0.116 to 1.112) | **0.016** | 1.957 (1.105 to 2.809) | **<0.001** |
| Hepatic Fat Content | -0.789 (-1.404 to -0.175) | **0.012** | -0.923 (-1.562 to -0.285) | **0.005** | NA | NA | NA | NA | NA | NA | NA | NA |
| CAP Score | -0.280 (-0.634 to 0.074) | 0.121 | -1.376 (-2.540 to -0.213) | **0.02** | NA | NA | NA | NA | NA | NA | NA | NA |
| Hepatic Steatosis Index (HSI) | -0.128 (-0.611 to 0.355) | 0.603 | 0.000 (-0.526 to 0.526) | 1 | NA | NA | NA | NA | NA | NA | NA | NA |

Bolded outcome is statistically significant, p<0.05

**Supplementary Material 6:** Changes in Fibrosis Markers

|  | **After SGLT2i Treatment** | | **Comparisons against Controls** | | **Comparisons against TZD** | | **Comparisons against Incretin-based Therapies** | | **Comparisons against Insulin-based Therapies** | | **Comparisons against Metformin** | |
| --- | --- | --- | --- | --- | --- | --- | --- | --- | --- | --- | --- | --- |
|  | **SMD (95% CI)** | **P-value** | **SMD (95% CI)** | **P-value** | **SMD (95% CI)** | **P-value** | **SMD (95% CI)** | **P-value** | **SMD (95% CI)** | **P-value** | **SMD (95% CI)** | **P-value** |
| FIB-4 Index | -0.152 (-0.450 to 0.146) | 0.318 | -0.384 (-0.787 to 0.018) | 0.061 | -0.780 (-1.281 to -0.279) | **0.002** | NA | NA | NA | NA | NA | NA |
| Liver Stiffness Measurement | -0.250 (-0.735 to 0.234) | 0.312 | -0.312 (-0.841 to 0.217) | 0.247 | NA | NA | NA | NA | NA | NA | NA | NA |
| NAFLD Fibrosis Score | -0.011 (-0.493 to 0.472) | 0.965 | -0.250 (-0.778 to 0.278) | 0.354 | NA | NA | NA | NA | NA | NA | NA | NA |
| Mac-2 Binding Protein | -0.051 (-0.534 to 0.431) | 0.835 | -0.166 (-0.693 to 0.361) | 0.537 | NA | NA | NA | NA | NA | NA | NA | NA |
| Hyaluronic Acid | 0.160 (-0.323 to 0.643) | 0.517 | 0.013 (-0.513 to 0.539) | 0.962 | NA | NA | NA | NA | NA | NA | NA | NA |
| Hematocrit | 0.317 (-0.169 to 0.802) | 0.201 | 0.284 (-0.245 to 0.812) | 0.292 | NA | NA | NA | NA | NA | NA | NA | NA |
| NAFIC Score | -0.569 (-1.062 to -0.077) | **0.023** | -0.692 (-1.233 to -0.15) | **0.012** | NA | NA | NA | NA | NA | NA | NA | NA |
| Serum Ferritin | -0.409 (-0.694 to -0.124) | **0.005** | -0.814 (-1.688 to 0.059) | 0.068 | -1.312 (-3.313 to 0.689) | 0.199 | NA | NA | -0.294 (-0.783 to 0.195) | 0.239 | NA | NA |

Bolded outcome is statistically significant, p<0.05

**Supplementary Material 7:** Changes in Liver and Renal Biomarkers

|  | **After SGLT2i Treatment** | | **Comparisons against Controls** | | **Comparisons against TZD** | | **Comparisons against Incretin-based Therapies** | | **Comparisons against Insulin-based Therapies** | | **Comparisons against Metformin** | |
| --- | --- | --- | --- | --- | --- | --- | --- | --- | --- | --- | --- | --- |
|  | **SMD (95% CI)** | **P-value** | **SMD (95% CI)** | **P-value** | **SMD (95% CI)** | **P-value** | **SMD (95% CI)** | **P-value** | **SMD (95% CI)** | **P-value** | **SMD (95% CI)** | **P-value** |
| Total Bilirubin | -0.333 (-0.929 to 0.262) | 0.272 | 0.000 (-0.606 to 0.606) | 1 | NA | NA | NA | NA | NA | NA | NA | NA |
| AST | -0.539 (-0.720 to -0.357) | **<0.001** | -0.421 (-0.680 to -0.161) | **0.001** | -0.232 (-0.576 to 0.112) | 0.186 | -0.274 (-0.600 to 0.051) | 0.098 | -0.686 (-1.186 to -0.185) | **0.007** | NA | NA |
| ALT | -0.633 (-0.892 to -0.373) | **<0.001** | -0.468 (-0.685 to -0.251) | **<0.001** | -0.287 (-1.038 to 0.464) | 0.454 | -0.329 (-0.824 to 0.165) | 0.192 | -0.551 (-1.046 to -0.055) | **0.029** | -0.466 (-1.169 to 0.236) | 0.193 |
| GGT | -0.330 (-0.530 to -0.129) | **0.001** | -0.295 (-0.834 to 0.243) | 0.283 | 0.450 (-0.444 to 1.344) | 0.324 | 0.112 (-0.475 to 0.698) | 0.709 | -0.639 (-1.138 to -0.140) | **0.012** | NA | NA |
| Albumin | 0.353 (0.034 to 0.671) | **0.03** | 0.363 (0.047 to 0.678) | **0.024** | NA | NA | NA | NA | NA | NA | NA | NA |
| Platelet Count | -0.051 (-0.364 to 0.261) | 0.747 | -0.039 (-0.755 to 0.677) | 0.916 | NA | NA | 0.648 (0.047 to 1.250) | **0.035** | NA | NA | NA | NA |
| eGFR | -0.120 (-0.539 to 0.298) | 0.573 | 0.063 (-0.266 to 0.391) | 0.708 | NA | NA | 0.445 (-0.148 to 1.038) | 0.142 | NA | NA | NA | NA |
| Uric Acid | -0.279 (-0.764 to 0.206) | 0.26 | -0.379 (-0.910 to 0.151) | 0.161 | NA | NA | NA | NA | NA | NA | NA | NA |

Bolded outcome is statistically significant, p<0.05

**Supplementary Material 8:** Quality Assessment of Included Studies

| Author, Year | Country | Study  Design | Selection | Comparability | Outcome | New-castle Ottawa Scale (NOS)  Total Score |
| --- | --- | --- | --- | --- | --- | --- |
| Ito et al 2017 | Japan | RCT | NA | NA | NA | -* |
| Seko et al 2017 | Japan | Retrospective Study | 3 | 2 | 2 | 7/9 |
| Choi et al 2018 | Korea | Retrospective Study | 3 | 0 | 2 | 5/9 |
| Kuchay et al 2018 | India | RCT | NA | NA | NA | -* |
| Shibuya et al 2018 | Japan | RCT | NA | NA | NA | -* |
| Aso et al 2019 | Japan | RCT | NA | NA | NA | -* |
| Shimizu et al 2019 | Japan | RCT | NA | NA | NA | -* |
| Han et al 2020 | Korea | RCT | NA | NA | NA | -* |
| Yano et al 2020 | Japan | Retrospective study | 2 | 1 | 2 | 5/9 |
| Kinoshita et al 2020 | Japan | RCT | NA | NA | NA | -* |

Legend:

-*Risk of Bias assessment was conducted for RCTs
